# HIV-1 infection and CD4 T cell depletion in the humanized Rag2^-/-^γc^-/- ^(RAG-hu) mouse model

**DOI:** 10.1186/1742-4690-3-76

**Published:** 2006-11-01

**Authors:** Bradford K Berges, William H Wheat, Brent E Palmer, Elizabeth Connick, Ramesh Akkina

**Affiliations:** 1 Department of Microbiology, Immunology, and Pathology, Colorado State University, Fort Collins, CO 80523, USA; 2 Department of Medicine, University of Colorado Health Sciences Center, Denver, CO 80262, USA; 3 Division of Infectious Disease, University of Colorado Health Sciences Center, Denver, CO 80262, USA

## Abstract

**Background:**

The currently well-established humanized mouse models, namely the hu-PBL-SCID and SCID-hu systems played an important role in HIV pathogenesis studies. However, despite many notable successes, several limitations still exist. They lack multi-lineage human hematopoiesis and a functional human immune system. These models primarily reflect an acute HIV infection with rapid CD4 T cell loss thus limiting pathogenesis studies to a short-term period. The new humanized Rag2^-/-^γc^-/- ^mouse model (RAG-hu) created by intrahepatic injection of CD34 hematopoietic stem cells sustains long-term multi-lineage human hematopoiesis and is capable of mounting immune responses. Thus, this model shows considerable promise to study long-term in vivo HIV infection and pathogenesis.

**Results:**

Here we demonstrate that RAG-hu mice produce human cell types permissive to HIV-1 infection and that they can be productively infected by HIV-1 ex vivo. To assess the capacity of these mice to sustain long-term infection in vivo, they were infected by either X4-tropic or R5-tropic HIV-1. Viral infection was assessed by PCR, co-culture, and in situ hybridization. Our results show that both X4 and R5 viruses are capable of infecting RAG-hu mice and that viremia lasts for at least 30 weeks. Moreover, HIV-1 infection leads to CD4 T cell depletion in peripheral blood and thymus, thus mimicking key aspects of HIV-1 pathogenesis. Additionally, a chimeric HIV-1 NL4-3 virus expressing a GFP reporter, although capable of causing viremia, failed to show CD4 T cell depletion possibly due to attenuation.

**Conclusion:**

The humanized RAG-hu mouse model, characterized by its capacity for sustained multi-lineage human hematopoiesis and immune response, can support productive HIV-1 infection. Both T cell and macrophage tropic HIV-1 strains can cause persistent infection of RAG-hu mice resulting in CD4 T cell loss. Prolonged viremia in the context of CD4 T cell depletion seen in this model mirrors the main features of HIV infection in the human. Thus, the RAG-hu mouse model of HIV-1 infection shows great promise for future in vivo pathogenesis studies, evaluation of new drug treatments, vaccines and novel gene therapy strategies.

## Background

Animal models played an important role in the understanding of HIV pathogenesis and in preclinical evaluation of therapeutic strategies [1-4]. In this regard, the severe combined immunodeficient C.B.-17 SCID/SCID (SCID) mouse model initially provided an in vivo system to study murine hemato-lymphoid differentiation and subsequently was further developed to investigate HIV pathogenesis [1,3,5-9]. Since these mice are immunodeficient, human cells and tissues can be transplanted without rejection. Two well-established mouse models have been used in various studies through the years, namely the hu-PBL-SCID and SCID-hu mouse models. The hu-PBL-SCID mouse model is created by injecting human peripheral blood mononuclear cells intraperitonially. Many elegant studies on HIV pathogenesis and passive immunity using monoclonal antibodies were conducted by Mosier and colleagues [3,5,6]. However, due to the lack of de novo development of continuously differentiating human cells, long-term studies on HIV pathogenesis are not possible in this system.

The second model, the SCID-hu mouse, is created by surgical engraftment of human fetal hematopoietic tissue, namely thymus and liver, under the kidney capsule of the SCID mouse [1,4]. Four to six months post-implantation, a conjoint organ (thy/liv) that resembles human thymus develops. For as long as one year, these grafts sustain T cell lymphopoiesis as a predominant feature. Since the SCID-hu mouse provides an in vivo setting for normal thymopoiesis and HIV preferentially infects CD4 T cells, this model has been extensively used to investigate AIDS pathogenesis in the context of a human lymphoid organ. Many pioneering studies were conducted by McCune and Zack's groups [7-9]. Early experiments have shown that infection kinetics follow a dose- and time-dependent course. Studies with drugs like AZT demonstrated the feasibility of in vivo drug testing and paved the way for other novel approaches [10,11]. Later investigations elaborated the detrimental effects of the infection on the various subpopulations of thymocytes as well as thymic non-T-cell elements like thymic epithelial cells [8,12]. Viral strain-specific differences were documented; additionally, the roles of HIV accessory proteins such as nef in virulence were ascertained [13,14]. Viral latency could be established in this model, thus further expanding its utility [15,16]. Although the kinetics of CD4 T cell loss differ, both the SCID-hu and hu-PBL-SCID mouse models support infection with either of the R5 and X4 HIV-1 viral strains [14]. In addition to pathogenesis studies, another innovative exploitation of the SCID-hu mouse model has been in gene therapy studies [17-20].

Despite these notable successes with the above in vivo humanized mouse models, several limitations still exist. Chief among these are that the scope of these models is primarily limited to the study of an acute HIV infection lasting only a few weeks due to the rapid decline of susceptible cell populations and a lack of continual multi-lineage hematopoiesis providing a constant supply of a wide spectrum of hematopoietic cells that HIV infects in the human. Furthermore, there is no functional human immune system operating in these models, thus limiting the study of viral pathogenic effects in the absence of an immune response and precluding immunity studies.

Newer humanized mouse models have recently emerged that can rectify the above limitations [21-25]. Prominent among these is the humanized Rag2^-/-^γc^-/- ^mouse model (hereafter referred to as RAG-hu) [26-31]. This consists of a double mutant mouse of an alymphoid phenotype with defects in the genes encoding recombinase activating gene 2 (Rag2) and common cytokine receptor gamma chain. The Rag mutation prevents normal maturation of T and B lymphocytes. Absence of functional receptors for IL-2, IL-7 and other cytokines prevents the expansion of lymphocytes, including that of NK cells which function to reject foreign grafts. Intravenous administration of human hematopoietic stem cells together with exogenous administration of human cytokines leads to a better engraftment rate. A recent breakthrough of even more extensive engraftment without exogenous cytokine administration has been achieved by Manz and colleagues [28]. Intrahepatic injection of human CD34 hematopoietic stem cells into conditioned neonatal mice led to superior and sustained engraftment resulting in de novo multi-lineage human hematopoiesis with the production of T cells, B cells and dendritic cells. Formation of structured primary and secondary lymphoid organs was seen with human cells engrafting in thymus, bone marrow, spleen and lymph nodes. Importantly, productive human immune responses were seen when engrafted mice were immunized with tetanus toxoid and infected with Epstein-Barr virus. Thus, this model is distinguished from the previous humanized mouse models by its capacity for multi-lineage human hematopoiesis and the presence of a functional human immune system. Therefore, the RAG-hu mouse model offers several advantages for HIV research.

Due to these unique features, we evaluated this new humanized mouse model for its susceptibility to HIV-1 infection and its utility for pathogenesis studies. In these proof-of-concept studies we show that RAG-hu mice are permissive to infection with both R5 and X4 tropic HIV-1, displaying prolonged viremia and CD4 T cell depletion characteristic of HIV infection and disease in the human.

## Results and discussion

### Hematopoietic cells differentiated in vivo in humanized Rag2^-/-^γc^-/- ^mice (RAG-hu mice) are susceptible to HIV-1 infection

**Figure 1:**
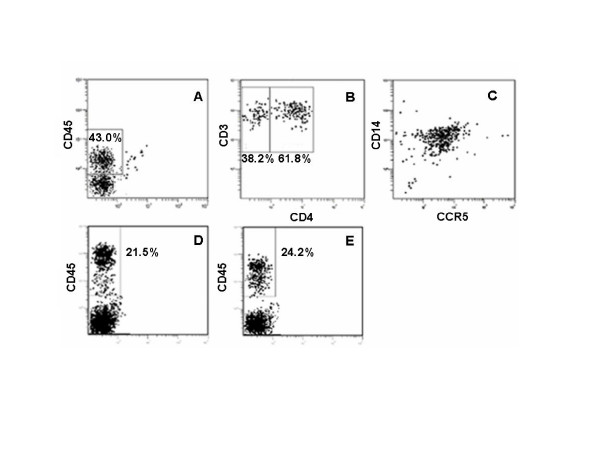
**Human cell engraftment in the peripheral blood of CD34 cell-reconstituted Rag2^-/-^γc^-/- ^mice and duration of engraftment**. Conditioned neonatal mice were injected with CD34 cells intrahepatically. At different times post-reconstitution, mice were bled to detect human cell engraftment. Peripheral blood cells were stained with different antibodies after RBC lysis and analyzed by FACS. (A) Cells stained with antibodies against the human panleukocyte marker CD45 at 12 weeks post-engraftment. (B) Cells stained with antibodies against the T cell markers CD3 and CD4. (C) Cells stained with antibodies against the monocyte markers CD14 and CCR5. To analyze the duration of engraftment, peripheral blood cells from an engrafted mouse were stained with antibodies against CD45 at 12 weeks (D) and 52 weeks (E) post-engraftment.

Previous studies of Traggiai et al established the multi-lineage human hematopoiesis in CD34 cell reconstituted Rag2^-/-^γc^-/- ^mice [28]. To systematically evaluate the utility of the RAG-hu mouse for HIV-1 infection studies, we first constructed RAG-hu mice by intrahepatic injection of human fetal liver-derived CD34^+ ^cells into conditioned neonatal BALB/c-Rag2^-/-^γc^-/- ^mice. Our initial experiments evaluated the transplanted mice to verify the levels of human cell engraftment, duration of their persistence, tissue distribution, and the presence of HIV-1 susceptible T cells and monocytes. Human cell engraftment was determined by FACS analysis of peripheral blood cells after staining with the human panleukocyte marker CD45 beginning 12 weeks post-injection. Over 150 mice have been evaluated to date. Of the mice showing detectable levels of engraftment (97%), the human cell levels ranged from 5–89%. Over 50% of mice showed >30% engraftment. A representative FACS plot depicting levels of engrafted CD45 cells is shown in Fig. 1A. We also determined the duration of engraftment and persistence of human cells in RAG-hu mice. When the engrafted mice were analyzed at one year post-engraftment, similar levels of human hematopoiesis could be seen relative to the levels seen at 12 weeks (Fig 1D and 1E).

To verify the presence of HIV-1 susceptible cells as well as human immune cells in the engrafted mice, we FACS analyzed peripheral blood cells to detect T cells and monocytes after staining with appropriate antibodies. T cell lineage populations staining positive for CD45/CD3^+/+ ^and CD45/CD3/CD4^+/+/+ ^T lymphocytes were detected in peripheral blood (Fig. 1B) as well as in thymus, spleen and lymph nodes (data not shown), similar to previous reports. Human monocytes were found in peripheral blood displaying characteristic CD45, CD14, and CCR5 markers (Fig. 1C), similar to the markers seen with macrophages originating from CD34 cells differentiated in vitro [32]. Monocytic cells were also seen in the lymph nodes and spleen (data not shown). We also assessed the CD4:CD3 cell ratio by FACS and found it to be similar (2.3:1) in all organs examined (data not shown) and at the high end of the normal human range [33]. With regard to the monocyte populations in the peripheral blood, the CD14^+ ^cells detected were predominantly of the CD14lo phenotype. The CD14lo population is typically associated with high CCR5 expression in human, which was also the case here in the RAG-hu mice.

**Figure 2:**
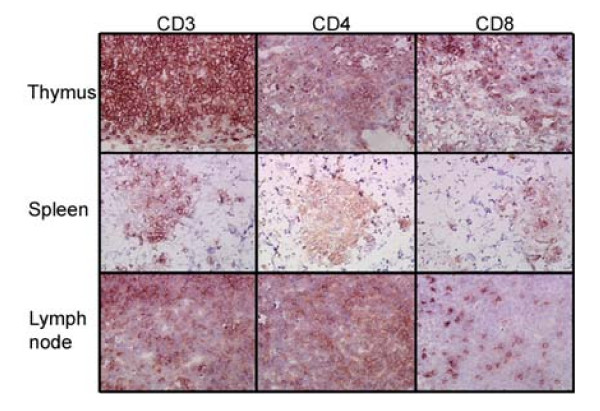
**Human T cell engraftment in lymphoid organs**. CD34 cell-reconstituted mice were sacrificed at 19 weeks post-engraftment, and thymus, spleen and lymph nodes were collected. Tissue sections were subjected to immuno-staining with different antibodies specific for human T cells as described in methods.

To further verify the presence of human T cells in lymphoid organs, an engrafted mouse was sacrificed and thymus, spleen, and lymph node sections were stained to detect the human T cell markers CD3, CD4, and CD8 (Fig. 2). Both CD4 and CD8 positive T cell sub-populations were detected in each of the three organs, with a high density of T cells present in the thymus and lymph nodes. T cells were seen as minor clusters in the spleen. These data collectively confirmed the successful engraftment of Rag2^-/-^γc^-/- ^mice with human CD34 hematopoietic progenitor cells and their lineage specific differentiation into HIV-1 susceptible human T cells and monocytes.

**Figure 3:**
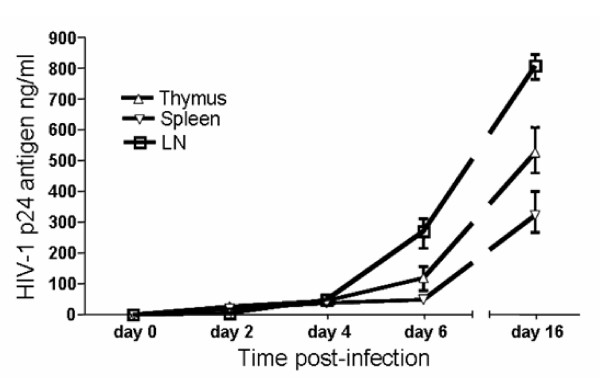
**Ex vivo productive HIV-1 infection in human cells differentiated in reconstituted Rag2^-/-^γc^-/- ^mice**. Thymus, spleen and lymph node tissues were collected at 16 weeks post-engraftment. Single cell suspensions were made and stimulated for 3 days with PHA and IL-2, and later challenged with HIV-1 NL4-3 HSA reporter virus. To detect productive viral infection, culture supernatants were analyzed by p24 ELISA at different days post-infection.

Before embarking on in vivo infections of these humanized mice, we first determined if the human cells matured in vivo were susceptible to HIV-1 infection ex vivo. Accordingly, cells obtained from lymphoid organs of RAG-hu mice, namely thymus, spleen and lymph nodes, were cultured in vitro and infected with a NL4-3 (X4-tropic) HIV-1 reporter virus that expresses the murine CD24 heat stable antigen (HSA). Our results showed productive infection of these cells as shown by increasing levels of HIV-1 p24 production at different days post-infection (Fig. 3). These data indicated that the engrafted CD34 cells matured into HIV-1 susceptible cells.

### RAG-hu mice are permissive for chronic HIV-1 infection

**Figure 4:**
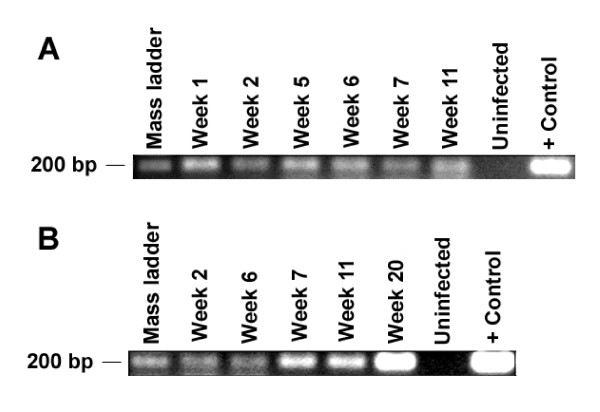
**PCR detection of HIV-1 in infected RAG-hu mice**. Peripheral blood was collected from infected mice at different weeks post-infection. Cellular and plasma fractions were separated by centrifugation. DNA from the cellular fractions was subjected to DNA PCR to detect integrated virus (A), whereas the RNA extracted from the plasma fraction was subjected to RT-PCR to detect cell-free virus (B). Results from a representative HIV-1 infected RAG-hu mouse (#16) are shown.

After establishing that RAG-hu mice generate differentiated human cells susceptible to HIV-1 infection, we next proceeded to evaluate if these mice can be productively infected in vivo. Mice were infected intraperitoneally with either HIV-1 X4 tropic NL4-3 (n = 9) or R5 tropic BaL (n = 5) viruses. In some experiments a HIV-1 strain with a GFP reporter gene (NLENG1-IRES) was used either alone (n = 2) or in combination with NL4-3 virus (n = 5) for infection to facilitate detection of infected cells in peripheral blood samples via FACS analysis. Blood samples were drawn roughly at weekly intervals, and the cellular and plasma fractions were separated. DNA PCR was used to detect integrated provirus and RT-PCR was performed to detect the circulating cell-free virus. A summary of both types of PCR analyses for viral detection is presented in Table 1 and a representative agarose gel showing the amplified PCR products is shown in Fig 4. Select plasma samples were also analyzed by Q-RT-PCR to determine HIV-1 viral load. In infected RAG-hu mice, the HIV-1 viral loads reached as high as 1.2 × 10^7 ^copies/ml (Table 1). However, not all samples could be evaluated due to insufficient sample volumes and/or the presence of a PCR inhibitor.

**Table 1:** Detection and quantification of HIV-1 in peripheral blood by PCR

***Experiment 1***					
Mouse	Infection	1 w	11 w	24 w	30 w

17	Ctrl	-	-	-	-
40	Ctrl	-	-	-	-
1	N+G	+	n/a	n/a	n/a
2	N+G	+	n/a	n/a	n/a
9	N+G	+ (369,000)^a^	+	+	+ (31,710)^b^
16	N+G	+ (165,000)^a^	+	+ (12,200,000)^a^	+ (81,440)^b^
21	G	+	+	+	+ (8,850)^b^
24	G	+	+	+	+ (17,220)^b^

***Experiment 2***					

Mouse	Infection	1 w	9 w	14 w	20 w

50	Ctrl	-	-	-	-
51	Ctrl	-	-	-	-
63	N	+	n/a	n/a	n/a
64	N	+	+ (28,000)^a^	n/a	n/a
65	N	+	n/a	n/a	n/a
70	N	+	n/a	n/a	n/a
56	N+G	+ (253,000)^a^	+	+ (52,250)^a^	n/a
71	B	+	+ (436,000)^a^	+	+ (261,140)^b^
72	B	+	+ (62,300)^a^	+	+ (480,770)^b^
75	B	+	+ (65,230)^b^	n/a	n/a
76	B	+	+ (430,280)^b^	n/a	n/a
88	B	+	+ (1,305,200)^b^	n/a	n/a

DNA PCR and RT-PCR analyses were performed on peripheral blood cells and plasma, respectively from infected RAG-hu mice using primers specific to the HIV-1 *pol *gene. Virus detection by one or both assays at various weeks post-infection is depicted as (+), while a lack of detection by both assays is depicted as such (-). Key: Ctrl = uninfected, N = NL4-3, G = NLENG1-IRES, B = BaL, n/a = available (sacrificed), ^a ^= viral load determined by Amplicor method, ^b ^= viral load determined with primers towards the LTR. Quantitative RT-PCR was performed on select samples to determine viral load per milliliter of plasma, as indicated in parentheses. Viral loads were initially determined with the Amplicor test (Roche Diagnostics). Later samples were analyzed with another primer set towards the HIV-1 LTR [41].

Evidence of virus could be detected by DNA- or RT-PCR in HIV-1 infected mice (n = 16) for up to 30 weeks post-infection, the longest time point examined. As expected, no PCR signal could be detected in either uninfected mice (n = 4), or unengrafted mice infected with HIV-1 BaL (n = 5). Weekly data was not collected for all the infected mice as some were sacrificed at an early time point. Similarly, 30 week data is not available for all the mice at this time as some mice were infected at a later date than the initial set. Although considerable variability was present in the level of human cell engraftment in individual mice at the time of infection, surprisingly, mice with as low as 5% engraftment (n = 3) were still able to support viremia. This indicates that infection could be sustained even in the context of modest human cell reconstitution.

The in vivo presence of virus in blood up to 30 weeks post-infection is suggestive of prolonged viremia, which is typical of the chronic HIV-1 infection seen in the human. In contrast, in the SCID-hu-PBL model of HIV-1 infection with the X4-tropic virus, viremia only lasted up to 3 weeks post-infection in most mice [34]. Persistent viremia in RAG-hu mice is most likely due to the continual generation and replenishment of target CD4 T cells from the engrafted hematopoietic stem cells, in contrast with that seen in SCID-hu-PBL mice in which virus-depleted cells are not replenished by endogenous production of T cells. We also determined whether RAG-hu mice support infection by R5 tropic HIV-1. Our results have shown productive infection in all the R5 BaL virus-injected mice. Thus, either X4 or R5 tropic HIV-1 can establish productive infection of RAG-hu mice akin to both the SCID-hu and SCID-hu-PBL mouse models. Although data was not generated for all time points, the available viral load data is indicative of high level of R5 virus replication.

To further confirm the PCR-based viremia data, virus re-isolation from some of the infected animals was performed. Peripheral blood cells obtained from three X4-infected mice (up to 20 weeks post-infection) and thymocytes and splenocytes from one X4-infected mouse (12 weeks post-infection) were co-cultured with susceptible SupT1 cells to amplify the virus. The co-cultures were positive for viral p24 production, thus confirming the presence of viable infectious virus in infected animals (data not shown).

As mentioned previously, in some experiments mice were infected with a GFP reporter HIV-1 to facilitate FACS-based identification of infected cells biopsied from virus-injected mice as described above. However, few GFP^+ ^cells were detected by FACS analysis in either peripheral blood or lymphoid organs of sacrificed mice (data not shown). Nevertheless, the GFP virus-infected mice were consistently viremic, thus indicating its replicative capacity in vivo. Failure to detect large numbers of GFP-positive cells in infected mice is possibly due to attenuation of this modified reporter virus as compared to the wild-type strain (see below).

**Figure 5:**
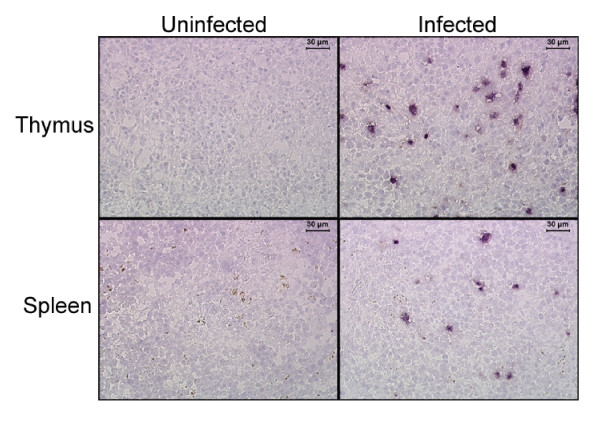
**Detection of HIV-1 in infected RAG-hu mouse tissues by in situ hybridization**. Thymus and spleen were collected at 12 weeks post-infection and sections were made from the frozen tissues. In situ hybridization was performed using digoxigenin-labeled antisense probes to detect HIV-1 RNA as described in methods. Dark staining cells indicate the presence of HIV-1.

To further confirm active HIV-1 replication in vivo, infected mice were sacrificed and lymphoid organs were analyzed for viral presence in histological sections. In situ hybridization using an HIV-specific probe was performed on tissue sections of spleen and thymus (Fig. 5). Infected cells were readily detected in mouse #64 (X4 infection), which was sacrificed at 12 weeks post-infection. Quantification of HIV-infected cells in these organs revealed 167 positive cells/mm^2 ^in thymus and 0.8 positive cells/mm^2 ^in spleen. As expected, no HIV-positive cells were detected in an uninfected mouse. These data indicated that HIV-infected cells are dispersed in various lymphoid organs as seen in the human.

### HIV-1 infection leads to CD4 T cell depletion in RAG-hu mice

A central hallmark of HIV infection is the gradual depletion of CD4 T lymphocytes, which are primary targets of HIV infection. To investigate if this phenomenon is recapitulated in HIV-1 infected RAG-hu mice, the levels of CD4 T cells in peripheral blood at different times post-infection were determined. Cells were stained for the pan-T cell marker CD3 as well as CD4, and the ratio of CD3^+^CD4^+ ^to CD3^+^CD4^- ^was used to measure depletion of CD4^+ ^T cells as described previously in hu-PBL-SCID mice HIV infection studies [35]. Baseline ratios in each mouse were established in pre-bleeds before infection (mean 70% CD4:CD3 ratio, range 50–85%, n = 24).

**Figure 6:**
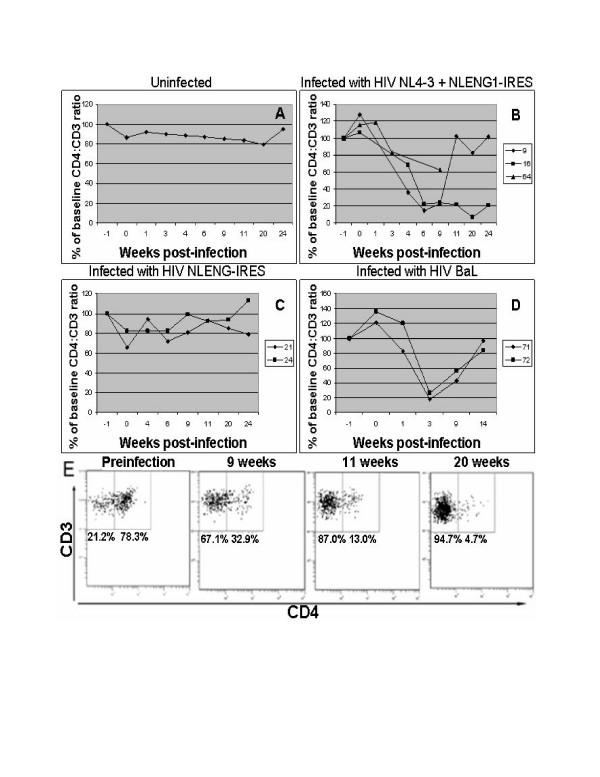
**CD4 T cell depletion in peripheral blood of HIV-1 infected RAG-hu mice**. Peripheral blood was collected at different weeks post-infection and cells were stained with CD3 and CD4 antibodies and FACS analyzed. To determine the levels of CD4 T cells in the whole T cell population (stained with the pan T cell marker CD3), CD4:CD3 ratios were determined as described in methods. To obtain a baseline CD4:CD3 level for each individual mouse prior to HIV-1 infection, mice were bled a minimum of two times before infection. CD4 T cell levels are depicted as a percent of individual mouse baseline levels recorded at 1 week pre-infection. Shown are mean uninfected mouse levels (A, n = 4), infection with HIV-1 NL4-3 + NLENG1-IRES (B), infection with HIV-1 NLENG1-IRES alone (C), and infection with HIV-1 BaL (D). Also shown (E) are representative FACS plots from mouse #16 from various time points post-infection indicating the CD3CD4^+/+ ^and CD3CD4^+/- ^populations used to calculate the values shown in A-D.

Five of seven infected mice exhibited depletion of CD4 T cells for at least 9 weeks (Fig. 6B–D), whereas none of the uninfected mice showed any CD4 T cell loss (Fig. 6A). CD4 T cell depletion was first detected at 3 weeks post-infection and in one case persisted through at least 24 weeks (mouse #16) as shown in Fig. 6B. On subsequent analysis at 30 weeks, this mouse continued to display CD4 T cell depletion at 6% of initial levels (data not shown). A representative FACS plot showing selective CD4 T cell loss over a 20 week time period for mouse #16 is shown in Fig. 6E. Interestingly, the only infected mice not displaying CD4 T cell depletion were those infected with the GFP reporter virus alone (n = 2) (Fig. 6C). In a different experiment where mice (n = 3) were infected with both cell-associated and cell-free GFP reporter virus, CD4 depletion also did not occur (data not shown). It is possible that the insertion of the foreign GFP gene into the NL4-3 genome may have resulted in attenuation of its virulence. Although capable of causing persistent infection as assessed by PCR, the GFP virus harboring additional genomic burden might not be robust enough to produce CD4 T cell depletion. We noted that some mice (#s 9, 71, 72) exhibited profound CD4 depletion at 6 and 9 weeks post-infection (to ~20% of initial values), followed by a rebound of CD4 cells to pre-infection levels. Mouse #16 displayed a more sustained CD4 T cell depletion at 6, 9, 11, 20, 24 and 30 weeks post-infection (up until the last time point evaluated). The differences and fluctuations in CD4 T cell depletion levels could be due to different levels of engraftment and/or the physiological status of each individual mouse. In any case, future evaluations using larger numbers of mice will ascertain possible reasons and mechanisms. CD4 T cell depletion was also observed in both mice infected with the R5-tropic strain BaL, similar to the results seen in SCID-hu-PBL mice (Fig. 6D) [35]. The continued CD4 T cell depletion through at least 9 weeks indicates that R5-tropic virus is also pathogenic in RAG-hu mice. In another ongoing experiment, 2 additional mice infected with BaL virus and 1 additional mouse infected with NL4-3 were also found to have CD4 T cell depletion to below 50% of normal at 9 weeks post-infection.

**Figure 7:**
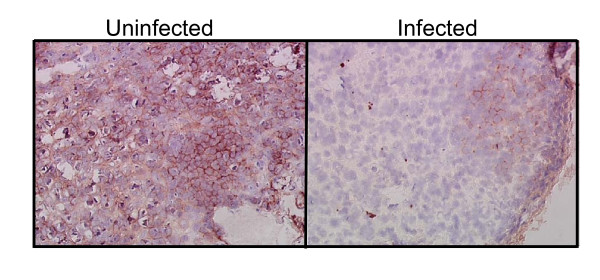
**Evidence for CD4 T cell depletion in HIV-1 infected RAG-hu mouse thymus**. Thymus was collected at 12 weeks post-infection (from mouse #64) and sections were made from frozen tissues. Tissue sections were subjected to immuno-staining with antibodies specific for human CD4 T cells as described in methods.

To further investigate CD4 T cell depletion at the infected tissue level, thymus from an uninfected control and infected mouse (same tissues as detailed above for HIV in situ hybridization) were evaluated by immuno-staining with CD4 antibodies (Fig. 7). Many CD4 cells were detected as expected in the uninfected thymus compared to that of infected thymus wherein there was a paucity of these cells. This data adds further evidence for CD4 T cell depletion as seen in peripheral blood assayed by FACS.

In a recent report Watanabe et al also demonstrated productive HIV-1 infection in a humanized mouse system (hNOG) capable of de novo multilineage human hematopoiesis using a different immunodeficient knock-out mouse on a NOD-SCID genetic background (NOD/SCID/IL2-R gamma chain knock-out mouse) [36]. Although only single time points for viral detection were shown in this report, high level viremia was seen in conjunction with CD4 T cell loss reflecting the results we have shown here. Humoral immune responses to HIV-1 antigens were also detected in some infected mice. Thus, these data from hNOG and RAG-hu mice corroborate that CD34 progenitor cell reconstituted mice with multilineage hematopoiesis are susceptible to HIV-1 infection. However, a number of parameters distinguish and differentiate between the hNOG and our present RAG-hu mouse models of HIV-1 infection. First, since the hNOG model uses mice with a NOD genetic background, this limits their experimental life span as these mice are prone to a high incidence of lymphomas and early death. On the contrary, the RAG-hu mouse has a normal life span, and sustains human hematopoiesis for more than a year. Second, in the hNOG model, HIV-1 infection could only be followed until 40 days (presumably due to their short life span) and thus the results obtained depict essentially an acute HIV-1 infection as seen in the previous SCID-hu and hu-PBL-SCID mouse models whereas in the RAG-hu system, the infection is more chronic, lasting 30 weeks (the latest time point analyzed to date, and likely to extend for more weeks pending further analysis). Third, although both X4 and R5 HIV-1 strains caused productive infection in hNOG mice, only the X4 virus infection resulted in CD4 T cell loss, whereas in the RAG-hu system both the viral strains caused CD4 T cell depletion. It is unclear why this difference exists given both the models are capable of multilineage hematopoiesis. Possible reasons could be due to the differences in mouse strains used, or alternatively, the R5 strains could also have displayed CD4 T cell depletion in hNOG mice if the infection was followed beyond 40 days. Overall, based on the comparisons above, the RAG-hu mouse model with its capacity for long-range hematopoiesis and chronic HIV-1 infection lasting beyond 30 weeks clearly offers several advantages over the hNOG model for long-term pathogenesis studies.

In summary, we have presented multiple lines of evidence demonstrating that RAG-hu mice support chronic HIV-1 infection with prolonged viremia when infected with either X4 or R5-tropic HIV-1 viral strains. The above proof-of-concept data also showed that viral infection leads to CD4 T cell depletion. Since prolonged viremia in the context of CD4 T cell depletion is seen in this model, many novel experiments are now possible. Different viral strains from the field can be evaluated for virulence and newer drugs can be tested for their long-term efficacy. In addition, the generation of drug resistant escape mutants can be evaluated during long-term treatment. Since the RAG-hu mice are shown to be immunocompetent, a thorough evaluation of their ability to generate HIV-specific humoral and cellular immune responses will be the next step to exploit this system for vaccine/immunity studies. Substantiating the potential for immune response to other antigens, recent results from our laboratory demonstrated Dengue virus infection and production of neutralizing antibody in RAG-hu mice (R. Troyer, J. Kuruvilla and R. Akkina; unpublished results to be reported elsewhere). Such experiments are currently underway to detect HIV immune responses. Furthermore, this model also permits systematic evaluation of anti-HIV gene therapeutic constructs expressed in differentiated T cells and macrophages originating from gene-transduced CD34 hematopoietic stem cells [18,37,38]. However, since this promising humanized mouse model is relatively new, many additional basic parameters of HIV-1 infection need to be vigorously established to realize its full potential in various future studies.

## Conclusion

RAG-hu mice reconstituted with human hematopoietic stem cells provide the unique features of multi-lineage human hematopoiesis and a functional human immune system which are ideal to study HIV pathogenesis in vivo. Here we showed that both T cell- and macrophage-tropic HIV-1 strains can cause persistent infection of RAG-hu mice resulting in CD4 T cell loss. Prolonged viremia in the context of CD4 T cell depletion seen in this model mirrors the main features of HIV infection in the human. The failure of a chimeric virus containing a reporter gene to cause CD4 T cell loss indicates possible attenuation in vivo. Thus, this new humanized mouse model of HIV-1 infection shows great promise as an in vivo experimental tool for the evaluation of new drug treatments, vaccines and gene therapies.

## Materials and methods

### Generation of humanized Rag2^-/-^γc^-/- ^mice (RAG-hu mice)

Humanized BALB/c-Rag2^-/-^γc^-/- ^mice were prepared essentially as described by Traggiai et al [28] with the exception that human fetal liver-derived CD34^+ ^cells were used for engraftment [17]. After irradiation at 350 rads, 2–3 day old neonatal mice were injected intrahepatically with 1 × 10^6 ^CD34^+ ^cells. Transplanted mice were screened for human cell engraftment at 12^+ ^weeks post-reconstitution. Peripheral blood was collected by tail bleed, and red blood cells were lysed using the Whole Blood Erythrocyte Lysing Kit (R&D Systems, Minneapolis, MN) per the manufacturer's protocol. The white cell fraction was stained with antibodies against the human pan-leukocyte marker CD45 and FACS analyzed to verify engraftment.

### CD34 cells and cell culture

Human fetal liver-derived CD34 cells were purified and cultured in cytokine media containing IL-3, IL-4 and SCF as described previously [17]. The SupT1 human lymphoid cell line was grown in RPMI 1640 (Invitrogen, Grand Island, NY) supplemented with 10% FBS. RAG-hu mouse-derived differentiated human hematopoietic cells were grown in Iscove's modified Dulbecco's medium (Invitrogen, Grand Island, NY) supplemented with 10% FBS, PHA and IL-2.

### HIV-1 infection of primary cells in vitro, viral co-culture and quantitation

Human cell reconstituted control or infected mice were sacrificed at different times post-reconstitution/infection. Tissues, namely bone marrow, thymus, liver, spleen, and mesenteric lymph nodes were collected and single suspensions were made followed by RBC lysis. Mononuclear cells (3 × 10^6^) were seeded and cultured as above. After 3 days, cells were infected with HIV-1 strain NL-r-HSAS (an X4-tropic strain expressing the murine heat stable antigen CD24) [9] at an MOI of 3. At 0, 2, and 6 days post-infection, supernatants were taken and assayed for productive infection by p24 ELISA (Beckman Coulter, Fullerton, CA). To isolate virus from infected mice by co-culture, 150 μl heparinized blood was taken by tail-bleed and red blood cells were lysed. The white blood cells were added to HIV-1 susceptible SupT1 cells in the presence of IL-2 and PHA, and supernatants were assayed for the presence of HIV p24 by ELISA (Beckman Coulter, Fullerton, CA).

### HIV-1 infection of humanized Rag2^-/-^γc^-/- ^mice

To infect human cell reconstituted RAG-hu mice, HIV-1 in a 100 μl volume was injected intraperitoneally at least 12 weeks after cell engraftment. Mice received either HIV-1 NL4-3 alone (1.2 × 10^5 ^i.u.), HIV-1 BaL alone (0.9 × 10^5 ^i.u.), HIV-1 NLENG1-IRES (NL4-3 engineered to express eGFP) [39] (1.6 × 10^5 ^i.u.), or a 1:1 mixture of HIV-1 NL4-3 and HIV-1 NLENG1-IRES at the same concentrations above. Mice were monitored daily.

### PCR analysis

To detect integrated HIV by PCR, DNA was extracted from a 25 μl cell pellet from peripheral blood using the QIAamp DNA Blood Kit (Qiagen, Valencia, CA). PCR was performed using *Taq *polymerase (Invitrogen, Carlsbad, CA) using a primer set to amplify a HIV-1 *pol *gene fragment as described previously [40]. PCR products were separated on a 2% agarose gel to resolve an expected 199-bp fragment. To detect cell-free HIV by RT-PCR, RNA was extracted from 10–70 μl of plasma using the QIAamp Viral RNA kit (Qiagen, Valencia, CA). cDNAs were produced with Superscript III reverse transcriptase (Invitrogen, Carlsbad, CA) and PCR amplified using HIV-1 *pol *primers [40]. Viral load in plasma samples was initially determined with the Amplicor test (Roche Diagnostics). Inadvertent use of heparin as an anticoagulant resulted in some Q-PCR inhibition. Later samples were collected without heparin and Q-RT-PCR was performed with another primer set towards the HIV-1 LTR [41].

### Flow cytometry

FACS analysis was used to assess human cell engraftment in peripheral blood and to detect various human hematopoietic cell populations in different lymphoid tissues. Whole blood was collected in heparinized capillary tubes then centrifuged to separate plasma from cells, and red blood cells were lysed as above. To analyze human cells in different lymphoid organs, single cell preparations were made followed by RBC lysis. Cell suspensions were stained with the following antibodies: hCD45-PE, (eBioscience), hCD4-PECy5, hCD3-PE, hCD14-PE (Caltag) and hCCR5 (BD Pharmingen). Stained cells were analyzed using a Coulter EPICS XL-MCL FACS analyzer (Beckman Coulter, Fullerton, CA). To measure CD4 T cell depletion in HIV-1 infected mice, they were tail-bled periodically. Blood cells were stained for CD3 and CD4 markers to determine CD3:CD4 cell ratios. CD4 T cell levels were calculated as a ratio of the entire CD3 population (CD4^+^CD3^+^:CD4^-^CD3^+^). To establish baseline CD4 T cell levels, all mice were analyzed twice before infection.

### In situ hybridization for HIV-1 RNA

In situ hybridization for HIV-1 RNA and quantification of frequencies of virus-producing cells was performed on 4-μm tissue sections from thymus and spleen previously frozen in OCT compound (VWR, Denver, CO) of one HIV-1-infected and one uninfected RAG-hu mouse, as previously described for human lymphoid tissues [42]. Three sections were evaluated from each tissue. In situ hybridization was performed using digoxigenin-labeled antisense riboprobes for env, gag, and nef, and detected using nitro blue tetrazolium/5-bromo-4-chloro-3-indoyl phosphate (NBT/BCIP). Sections were lightly counterstained with hematoxylin to enable visualization of nuclei. The number of virus-producing cells was determined by visual inspection and manually counting the positively stained cells on each section. The sum of the total number of virus-producing cells was divided by the total area of the tissue sections inspected, as quantified by a computerized image analysis system (Leica Q5001W Image Analysis, Leica, Cambridge, UK) to determine the number of HIV-1-producing cells per mm^2 ^tissue.

### Immuno-staining for lymphocyte and macrophage markers

Immuno-staining for human CD3 (Clone UCHT1, BD Pharmingen, San Diego, CA), CD4 (Clone RPA-T4, BD Pharmingen), and CD8 (Clone HIT8a, BD Pharmingen) was performed on 4-μm tissue sections from a RAG-hu mouse thymus, spleen, and lymph node previously frozen in OCT compound. Sections were initially fixed in 1% paraformaldehyde for 20 min., washed in Tris Buffered Saline (TBS; 0.05 M Tris hydrochloride, 0.15 M NaCl, pH 7.6) and treated with 1% hydrogen peroxide (Sigma, St. Louis, MO) in TBS to block endogenous peroxidase activity. Sections were further blocked for 15 min. with Avidin (Vector Laboratories, Burlingame, CA), washed in TBS, followed by Biotin (Vector Labs). Sections were stained using Vector Laboratories' Mouse on Mouse™ staining kit, according to the manufacturer's instructions. Color detection was performed with horseradish peroxidase streptavidin (Vector Laboratories) with NovaRed (Vector Laboratories) added as a substrate producing a red-brown color. Sections were lightly counterstained with hematoxylin like above to enable visualization of nuclei. Control sections were treated with antibody diluent and processed in parallel.

## Abbreviations

Acquired Immunodeficiency Syndrome (AIDS)

FACS (fluorescence activated cell sorting)

γ_c _(common gamma chain receptor)

GFP (green fluorescent protein)

HLA (human leukocyte antigen)

HSA (heat stable antigen)

HSC (hematopoietic stem cell)

Human Immunodeficiency Virus (HIV)

NK (natural killer)

PBL (peripheral blood leukocytes)

PCR (polymerase chain reaction)

PHA (phytohaemagglutinin)

Q-PCR (quantitative polymerase chain reaction)

Q-RT-PCR (quantitative reverse transcriptase polymerase chain reaction)

R5 (CCR5)

Rag2 (recombinase activating gene 2)

RT-PCR (reverse transcriptase polymerase chain reaction)

X4 (CXCR4)

SCID (severe combined immunodeficiency)

## Competing interests

The author(s) declare that they have no competing interests.

## Authors' contributions

BB and WW prepared RAG-hu mice, performed HIV infections and FACS analysis.

BB was also responsible for PCR assays. BP performed the multiparametric FACS analysis. EC contributed to immuno-histology and in situ hybridizations. RA was responsible for the conception, overall experimental design and implementation of the project. All authors read and approved the final manuscript.

## References

[B1] McCune JM, Namikawa R, Kaneshima H, Shultz LD, Lieberman M, Weissman IL (1988). The SCID-hu mouse: murine model for the analysis of human hematolymphoid differentiation and function. Science.

[B2] Mosier DE, Gulizia RJ, Baird SM, Wilson DB, Spector DH, Spector SA (1991). Human immunodeficiency virus infection of human-PBL-SCID mice.. Science.

[B3] Mosier DE (1996). Human immunodeficiency virus infection of human cells transplanted to severe combined immunodeficient mice. Adv Immun.

[B4] Jamieson BD, Aldrovandi GM, Zack JA (1996). The SCID-hu mouse: an in-vivo model for HIV-1 pathogenesis and stem cell gene therapy for AIDS. Sem in Immun.

[B5] Mosier DE, Gulizia RJ, Baird SM, Spector S, Spector D, Kipps TJ, Fox RI, Carson DA, Cooper N, Richman DD, al. (1989). Studies of HIV infection and the development of Epstein-Barr virus-related B cell lymphomas following transfer of human lymphocytes to mice with severe combined immunodeficiency. Curr Top Micro Immun.

[B6] Mosier DE, Gulizia RJ, MacIsaac PD, Corey L, Greenberg PD (1993). Resistance to human immunodeficiency virus 1 infection of SCID mice reconstituted with peripheral blood leukocytes from donors vaccinated with vaccinia gp160 and recombinant gp160.. Proc Natl Acad Sci USA.

[B7] Bonyhadi ML, Rabin L, Salimi S, Brown DA, Kosek J, McCune JM, Kaneshima H (1993). HIV induces thymus depletion in vivo. Nature.

[B8] Aldrovandi GM, Feuer G, Gao L, Jamieson B, Kristeva M, Chen IS, Zack JA (1993). The SCID-hu mouse as a model for HIV-1 infection. Nature.

[B9] Jamieson BD, Zack JA (1998). In vivo pathogenesis of a human immunodeficiency virus type 1 reporter virus. J Virol.

[B10] McCune JM, Namikawa R, Shih CC, Rabin L, Kaneshima H (1990). Suppression of HIV infection in AZT-treated SCID-hu mice. Science.

[B11] Stoddart CA, Moreno ME, Linquist-Stepps VD, Bare C, Bogan MR, Gobbi A, Buckheit RWJ, Bedard J, Rando RF, McCune JM (2000). Antiviral activity of 2'-deoxy-3'-oxa-4'-thiocytidine (BCH-10652) against lamivudine-resistant human immunodeficiency virus type 1 in SCID-hu Thy/Liv mice.. Antimicrob Agents Chemother.

[B12] Stanley SK, McCune JM, Kaneshima H, Justement JS, Sullivan M, Boone E, Baseler M, Adelsberger J, Bonyhadi M, Orenstein J (1993). Human immunodeficiency virus infection of the human thymus and disruption of the thymic microenvironment in the SCID-hu mouse. J Exp Med.

[B13] Jamieson BD, Zack JA (1999). Murine models for HIV disease. AIDS.

[B14] Mosier DE (2000). Human xenograft models for virus infection. Virol.

[B15] Brooks DG, Hamer DH, Arlen PA, Gao L, Bristol G, Kitchen CM, Berger EA, Zack JA (2003). Molecular characterization, reactivation, and depletion of latent HIV. Immunity.

[B16] Arlen PA, Brooks DG, Gao LY, Vatakis D, Brown HJ, Zack JA (2006). Rapid expression of human immunodeficiency virus following activation of latently infected cells. J Virol.

[B17] Akkina RK, Rosenblatt JD, Campbell AG, Chen IS, Zack JA (1994). Modeling human lymphoid precursor cell gene therapy in the SCID-hu mouse. Blood.

[B18] Banerjea A, Li MJ, Bauer G, Remling L, Lee NS, Rossi J, Akkina R (2003). Inhibition of HIV-1 by lentiviral vector-transduced siRNAs in T lymphocytes differentiated in SCID-hu mice and CD34+ progenitor cell-derived macrophages.. Mol Ther.

[B19] Bai J, Banda N, Lee NS, Rossi J, Akkina R (2002). RNA-based anti-HIV-1 gene therapeutic constructs in SCID-hu mouse model.. Mol Ther.

[B20] Banerjea A, Li MJ, Remling L, Rossi J, Akkina R (2004). Lentiviral transduction of Tar Decoy and CCR5 ribozyme into CD34+ progenitor cells and derivation of HIV-1 resistant T cells and macrophages.. AIDS Res Ther.

[B21] Goldman JP, Blundell MP, Lopes L, Kinnon C, Di Santo JP, Thrasher AJ (1998). Enhanced human cell engraftment in mice deficient in RAG2 and the common cytokine receptor gamma chain. Br J Haematol.

[B22] Shultz LD, Lyons BL, Burzenski LM, Gott B, Chen X, Chaleff S, Kotb M, Gillies SD, King M, Mangada J, Greiner DL, Handgretinger R (2005). Human lymphoid and myeloid cell development in NOD/LtSz-scid IL2R gamma null mice engrafted with mobilized human hemopoietic stem cells. J Immun.

[B23] Yahata T, Ando K, Nakamura Y, Ueyama Y, Shimamura K, Tamaoki N, Kato S, Hotta T (2002). Functional human T lymphocyte development from cord blood CD34+ cells in nonobese diabetic/Shi-scid, IL-2 receptor gamma null mice. J Immun.

[B24] Ishikawa F, Yasukawa M, Lyons B, Yoshida S, Miyamoto T, Yoshimoto G, Watanabe T, Akashi K, Shultz LD, Harada M (2005). Development of functional human blood and immune systems in NOD/SCID/IL2 receptor {gamma} chain(null) mice. Blood.

[B25] Hiramatsu H, Nishikomori R, Heike T, Ito M, Kobayashi K, Katamura K, Nakahata T (2003). Complete reconstitution of human lymphocytes from cord blood CD34+ cells using the NOD/SCID/gammacnull mice model. Blood.

[B26] Kirberg J, Berns A, von Boehmer H (1997). Peripheral T cell survival requires continual ligation of the T cell receptor to major histocompatibility complex-encoded molecules. J Exp Med.

[B27] Gimeno R, Weijer K, Voordouw A, Uittenbogaart CH, Legrand N, Alves NL, Wijnands E, Blom B, Spits H (2004). Monitoring the effect of gene silencing by RNA interference in human CD34+ cells injected into newborn RAG2-/- gammac-/- mice: functional inactivation of p53 in developing T cells. Blood.

[B28] Traggiai E, Chicha L, Mazzucchelli L, Bronz L, Piffaretti JC, Lanzavecchia A, Manz MG (2004). Development of a human adaptive immune system in cord blood cell-transplanted mice.. Science.

[B29] Macchiarini F, Manz MG, Palucka AK, Shultz LD (2005). Humanized mice: are we there yet?. J Exp Med.

[B30] Chicha L, Tussiwand R, Traggiai E, Mazzucchelli L, Bronz L, Piffaretti JC, Lanzavecchia A, Manz MG (2005). Human Adaptive Immune System Rag2-/-{gamma}c-/- Mice.. Ann N Y Acad Sci.

[B31] Weijer K, Uittenbogaart CH, Voordouw A, Couwenberg F, Seppen J, Blom B, Vyth-Dreese FA, Spits H (2002). Intrathymic and extrathymic development of human plasmacytoid dendritic cell precursors in vivo. Blood.

[B32] Anderson JS, Bandi S, Kaufman DS, Akkina R (2006). Derivation of normal macrophages from human embryonic stem (hES) cells for applications in HIV gene therapy. Retrovirology.

[B33] Hulstaert F, Hannet I, Deneys V, Munhyeshuli V, Reichert T, De Bruyere M, Strauss K (1994). Age-related changes in human blood lymphocyte subpopulations. II. Varying kinetics of percentage and absolute count measurements. Clin Immun Immunopath.

[B34] Picchio GR, Gulizia RJ, Wehrly K, Chesebro B, Mosier DE (1998). The cell tropism of human immunodeficiency virus type 1 determines the kinetics of plasma viremia in SCID mice reconstituted with human peripheral blood leukocytes. J Virol.

[B35] Gulizia RJ, Levy JA, Mosier DE (1996). The envelope gp120 gene of human immunodeficiency virus type 1 determines the rate of CD4-positive T-cell depletion in SCID mice engrafted with human peripheral blood leukocytes.. J Virol.

[B36] Watanabe S, Terashima K, Ohta S, Horibata S, Yajima M, Shiozawa Y, Dewan MZ, Yu Z, Ito M, Morio T, Shimizu N, Honda M, Yamamoto N (2006). Hematopoietic stem cell-engrafted NOD/SCID/IL2R{gamma}null mice develop human lymphoid system and induce long-lasting HIV-1 infection with specific humoral immune responses. Blood.

[B37] Akkina R, Banerjea A, Bai J, Anderson J, Li MJ, Rossi J (2003). siRNAs, ribozymes and RNA decoys in modeling stem cell-based gene therapy for HIV/AIDS.. Anticancer Research.

[B38] Anderson J, Banerjea A, Akkina R (2003). Bispecific short hairpin siRNA constructs targeted to CD4, CXCR4, and CCR5 confer HIV-1 resistance.. Oligonucleotides.

[B39] Kutsch O, Benveniste EN, Shaw GM, Levy DN (2002). Direct and quantitative single-cell analysis of human immunodeficiency virus type 1 reactivation from latency. J Virol.

[B40] Desire N, Dehee A, Schneider V, Jacomet C, Goujon C, Girard P, Rozenbaum W, Nicolas JC (2001). Quantification of human immunodeficiency virus type 1 proviral load by a TaqMan real-time PCR assay.. J Clin Microbiol.

[B41] Rouet F, Ekouevi DK, Chaix ML, Burgard M, Inwoley A, Tony TD, Danel C, Anglaret X, Leroy V, Msellati P, Dabis F, Rouzioux C (2005). Transfer and evaluation of an automated, low-cost real-time reverse transcription-PCR test for diagnosis and monitoring of human immunodeficiency virus type 1 infection in a West African resource-limited setting.. J Clin Microbiol.

[B42] Folkvord JM, Armon C, Connick E (2005). Lymphoid follicles are sites of heightened human immunodeficiency virus type 1 (HIV-1) replication and reduced antiretroviral effector mechanisms. AIDS Res Hum Retroviruses.

